# Integrating genome-wide association study into genomic selection for the prediction of agronomic traits in rice (*Oryza sativa* L.)

**DOI:** 10.1007/s11032-023-01423-y

**Published:** 2023-11-13

**Authors:** Yuanyuan Zhang, Mengchen Zhang, Junhua Ye, Qun Xu, Yue Feng, Siliang Xu, Dongxiu Hu, Xinghua Wei, Peisong Hu, Yaolong Yang

**Affiliations:** 1 Zhejiang Lab, Hangzhou, 311121 China; 2 CNRRI-Zhejiang Lab Computational Breeding Joint Laboratory, China National Rice Research Institute, Hangzhou, China; 3 National Nanfan Research Institute (Sanya), Chinese Academy of Agricultural Sciences, Sanya, 572024 China

**Keywords:** Genotype-to-phenotype, Statistical models, Genome-wide association study, Predictive ability

## Abstract

Accurately identifying varieties with targeted agronomic traits was thought to contribute to genetic selection and accelerate rice breeding progress. Genomic selection (GS) is a promising technique that uses markers covering the whole genome to predict the genomic-estimated breeding values (GEBV), with the ability to select before phenotypes are measured. To choose the appropriate GS models for breeding work, we analyzed the predictability of nine agronomic traits measured from a population of 459 diverse rice varieties. By the comparison of eight representative GS models, we found that the prediction accuracies ranged from 0.407 to 0.896, with reproducing kernel Hilbert space (RKHS) having the highest predictive ability in most traits. Further results demonstrated the predictivity of GS is altered by several factors. Moreover, we assessed the method of integrating genome-wide association study (GWAS) into various GS models. The predictabilities of GS combined peak-associated markers generated from six different GWAS models were significantly different; a recommendation of Mixed Linear Model (MLM)-RKHS was given for the GWAS-GS-integrated prediction. Finally, based on the above result, we experimented with applying the *P*-values obtained from optimal GWAS models into ridge regression best linear unbiased prediction (rrBLUP), which benefited the low predictive traits in rice.

**Supplementary Information:**

The online version contains supplementary material available at 10.1007/s11032-023-01423-y.

## Introduction

As one of the most important staple food crops for the population throughout the world, rice (*Oryza sativa* L.) is a key component of strategies aimed at ensuring sustainable food production (Muthayya et al. 2014). The priority of rice breeding is to select varieties with ideal traits such as high yield, resistance to stress, or other attractive properties. The conventional breeding method is to select ideal offspring with the aim to improve target traits by observing the phenotypes directly, but the efficiency is decreased by the interaction between gene and environment, errors of measurement, and other limitations (Zhao et al. 2022). Furthermore, the process is both time-consuming and labor-intensive. With advances in molecular technologies, genetic variation across the whole genome can be effectively captured, which has laid the foundation for molecular crop breeding. Initially, marker-assisted selection (MAS) was proposed to select individuals with quantitative trait locus (QTL)-associated markers (Bernardo 2008), but it is less suitable for traits controlled largely by polygenes with small effects (Riedelsheimer et al. 2012). To overcome the disadvantages of MAS, a new alternative to traditional MAS called genomic selection (GS) was proposed (Meuwissen et al. 2001).

GS was introduced in 2001 by Meuwissen et al. (2001) to estimate the effects of all loci and thus calculate the genomic-estimated breeding values (GEBV) of each individual. The basic process of GS starts with the creation of a training set and testing set. A training set with known genotype and phenotype information is used to model the association between genotype and phenotype; the developed model is then used to predict the phenotypic value of the testing set that has been genotyped only (Crossa et al. 2017). Research showed that prediction accuracy achieved using GS was superior to MAS (Heffner et al. 2011), and GS has wider employment in plant breeding (Budhlakoti et al. 2022). GS has proven its role in corn, wheat, rice, and many other crops (Marulanda et al. 2016; Meena et al. 2022). It can also predict the performance of natural and hybrid populations (Xu et al. 2014). In addition, GS can be used for the prediction of multiple agronomic traits including growth and even biological stresses (Desta and Ortiz 2014; Cooper et al. 2019). The major advantage of using GS is that it greatly increases the efficiency of breeding due to early selection. Based on the predicted GEBV, breeders do not have to wait until all the phenotypes are collected to screen for varieties of interest during phenotypic evaluations at different crop stages (Yu et al. 2016; Crossa et al. 2017). In this way, GS can greatly shorten the breeding cycle and reduce the cost of selection processes, thereby subsequently accelerating genetic gain (Xu et al. 2018; Fu et al. 2022). It can open new avenues for managing breeding programs in precise and intelligent ways (Xu et al. 2022).

Numerous statistical models, including parametric and non-parametric models, have been developed for GS to predict phenotypes using large sets of genetic markers (Howard et al. 2014). Parametric models mainly include best linear unbiased prediction (BLUP) (Habier et al. 2013), Bayesian models (González-Recio and Forni 2011), and least absolute shrinkage selection operator (LASSO); non-parametric models include random forest (RF), support vector machine (SVM), and reproducing kernel Hilbert space (RKHS) (De los Campos et al. 2010﻿). BLUP is one of the most widely used models in plant GS studies; it is a mixed model–based whole-genome regression approach that is used to estimate the marker effects (Habier et al. 2013). There are many variants of BLUP, such as genomic BLUP (GBLUP) (VanRaden 2008), ridge regression BLUP (rrBLUP) (Endelman 2011), and single-step GBLUP (ssGBLUP). GBLUP uses the genomic relationship matrix (G matrix) to predict phenotypes (VanRaden 2008). For rrBLUP, it assumes that all the markers have equal variances and small but non-zero effects; it is an efficient model for solving the over-parameterization problem in linear models (Endelman 2011). Combining the G matrix and additive relationship matrix, ssGBLUP can also achieve the aim of constructing a genotype-to-phenotype relationship. Unlike GBLUP and rrBLUP, most Bayesian models allow different markers to have diverse effects and variances (Habier et al. 2011). There are several variants of Bayesian models, such as BayesA, BayesB, BayesC, BayesCπ, Bayesian LASSO (BL), and Bayesian ridge regression (BRR) (Hoerl and Kennard 2000; de los Campos et al. 2009; Pérez et al. 2010). The varieties of Bayesian models differ in the prior distribution. As for RKHS, it is a mixed model that combines an additive genetic model with a kernel function, which is more effective for capturing non-additive effects (Gianola et al. 2006).

Achieving accurate prediction is essential for implementing GS approaches in plant breeding. Prediction accuracy is the standard measure for the usefulness of GS models. There are many methods for the validation of prediction ability (Xu 2017), but cross-validation (CV) is a typically evaluated method. In a K-fold CV, data is randomly divided into K equal parts, and each part is predicted once using parameters estimated from the other K-1 parts. The validation result is affected by various factors (Azodi et al. 2019). For instance, using various statistical models, studies indicated that there was a significant difference in predictability (Cruz et al. 2021). In addition, several researchers have illuminated that the predictive ability grows as the marker number increases until it reaches a plateau (Onogi et al. 2015). Other factors like the sample size of the training set, heritability (Yao et al. 2018), environment (Montesinos-López et al. 2016), and the span of linkage disequilibrium also require careful consideration. Currently, there are no models that can fit all the data well; thus, evaluation of the data of interest is necessary to select the best model with the maximum accuracy.

In reality, the implementation of GS in plant breeding is still in the early stages with many difficulties. Overfitting and underfitting are two major issues occurring when training models. If the phenomenon of overfitting happens, the trained model is likely to accurately predict the targets in the training dataset but fails when generating the prediction results of the test set. Using large amounts of markers results in the number of markers (*p*) vastly exceeding the number of observations (*n*), which creates the “curse of dimensionality” of data, causing the overfitting of a model (Schmidt et al. 2016; Crossa et al. 2017). Researchers have found that using a subset of significant markers can be an alternative for dealing with the large “*p*” and small “*n*” problem, but it is a challenging task to choose optimal markers with high predictability for the phenotype (Jeong et al. 2020). Genome-wide association study (GWAS) is a method for identifying single nucleotide polymorphism (SNP) markers associated with phenotype, and it has been utilized for genomic prediction studies. A new GS model termed GS + de novo GWAS that combines rrBLUP with markers selected from the results of GWAS on training data was invented (Spindel et al. 2016). In the same way, based on GWAS, a novel tool called GMStool for selecting optimal marker sets and predicting quantitative phenotypes was presented (Jeong et al. 2020). In addition, several studies have demonstrated the feasibility of combining GWAS results with GS, and there are numerous benefits to this approach (Zhang et al. 2014; Yilmaz et al. 2021). In fact, there are a variety of GWAS models that can be chosen. When incorporating GWAS into GS, some researchers use the model of Settlement of MLM Under Progressively Exclusive Relationship (SUPER) (Li et al. 2018), while others use Bayesian information and Linkage-disequilibrium Iteratively Nested Keyway (BLINK), Mixed Linear Model (MLM), Fixed and random model Circulating Probability Unification (FarmCPU), etc. (Rice and Lipka 2019; Tan and Ingvarsson 2022; Zhang et al. 2023). But, under the same genetic background, the results of a GWAS model applied to the GS effect optimal have not been studied and discussed.

Apart from the above insufficiency, there are still limited studies applying GS to rice breeding practice, especially when compared to other major crops such as maize and wheat, and it is currently unclear how to better utilize the GWAS results to improve prediction in rice. Consequently, it is necessary to explore appropriate methods which could potentially result in improved predictive ability in rice. The objectives of this study were to (1) compare the predictability of several GS models for different traits in the rice population, (2) test the performance of GS based on some affecting factors, (3) clarify the optimal model of integrating GWAS models into GS, and (4) further investigate the feasibility of incorporating weight matrix from the result of GWAS to improve predictability. Through this study, we proposed a breeding strategy to predict the best-performing candidates and select ideal varieties for rice improvement. Giving a reference way to further accelerate the process of rice breeding and reduce the breeding cost.

## Materials and methods

### Plant materials and phenotyping

The plant materials used in the present study were constructed from 469 rice germplasm accessions, which were collected from around 20 major rice-growing countries and contained abundant genetic diversity and phenotypic variation. Names and origins of the population were presented by Lu et al. (2015). For phenotyping, the rice lines in trials were grouped into two locations in 2014, Hangzhou (HZ), Zhejiang province, and LingShui (LS), Hainan province, using randomized complete block designs with three replications. Nine traits were analyzed, including (1) tiller number (TN), (2) tiller angle (TA), (3) plant height (PH), (4) flag leaf length (FLL), (5) flag leaf width (FLW), (6) the ratio of flag leaf length and flag leaf width (FLLW), (7) flag leaf angle (FLA), (8) panicle number (PN), and (9) panicle length (PL); the measurement methods of traits were elaborated by Lu et al. (2015). The best linear unbiased estimate (BLUE) values were computed for further analysis using a mixed linear model in the R package “lme4” (Bates et al. 2015); the values were standardized when the prediction models were trained.

### Genotypic dataset

A total of 5291 SNPs were extracted from 469 accessions in 2014 for details on sequencing, as described by Lu et al. (2015). Quality control of markers included the elimination of those SNPs with a minor allele frequency of lower than 5%, missing genotypes above 3%, and heterozygosity deviating from three standard deviations. Finally, 459 accessions with 3955 SNPs remained after this filtering. SNP genotype at each locus was coded either as − 1, 0, and 1 or 0, 1, and 2 using the analysis of GS and GWAS.

### Statistical methods

Eight models that represent different types of statistical methodologies were tested for estimating GEBV, including GBLUP, rrBLUP, BayesA, BayesB, BayesC, BL, BRR, and RKHS. GBLUP uses the G matrix in place of the traditional numerator pedigree-derived relationship matrix (VanRaden 2008). The G matrix can be obtained by

$$G=\frac{Z{Z}^{T}}{2{\sum }_{i=1}^{m}pi(1-pi)}$$

Here, *Z* is centralized through the subtraction of *P* from *M* which is an incidence matrix coded as − 1, 0, 1, the number of rows equal to the number of individuals and the number of columns equal to the number of markers. *Z*^*T*^ is the transpose matrix of *Z*. The minor allele frequency at locus *i* is *p*_*i*_ and column *i* of *P* is 2(*p*_*i*_ − 0.5). GBLUP and Bayesian model treat the effect of the label as a random effect; their model can be described as

$$y =\mathrm{ X}\beta +\mathrm{ Z}\alpha +\upvarepsilon$$

where *y* is a vector for *n* observations; *X* is a design matrix corresponding to *β*; *β* is a non-genetic vector of fix effects; *Z* is an *n* × *k* genotype covariates matrix; *k* is the genotypic indicators for individual *i* (where *i* = 1, 2,…, *n*) coded as − 1, 0, and 1; *α* is an *n* × 1 vector of random effects, and ε is a residual vector. The difference between models is mainly due to the prior distributions of *α*. All the unknown parameters were calculated by the restricted maximum likelihood method; the log-likelihood function is

$$L\left(\lambda\right)=-\frac12\ln\vert V\vert-\frac12(y-X\beta)^TV^{-1}(y-X\beta)-\frac12\ln\vert X^TV^{-1}X\vert$$

The solution of λ was obtained by maximizing the above likelihood function using the Newton iteration algorithm. λ represents the collection of unknowns, and the variance matrix is rewritten as *V*. Analyses were performed in R (R Core Team 2021) with the R packages sommer (Covarrubias-Pazaran 2016).

Bayesian estimates are calculated based on the Markov chain Monte Carlo method (MCMC), which is a computationally efficient method that produces full conditional distributions of the parameters. A variety of prior densities, from flat priors to priors that induce different types of shrinkage were chosen, which in turn produce different degrees of compression. The scaled-*t* density is the prior used in model BayesA, which assumes that the effect of the *i*th marker follows a normal distribution; the variance of the normal distribution follows the scaled inverse chi-squared distribution:${{\upsigma }_{\mathrm{i}}^{2}\sim \mathcal{X}}^{-2}(\mathrm{v},{\mathrm{S}}^{2})$. Besides, there implements two finite mixture priors: a mixture of a point of mass at zero and a Gaussian slab, named BayesC (Habier et al. 2011), and a mixture of a point of mass at zero and a scaled-t slab, known as BayesB (Meuwissen et al. 2001). The variance of BayesC is shared among markers, and that of BayesB is not. Park and Casella investigated a model that combined the Bayesian approach with LASSO called BL, allowing heterogeneity among markers, with some markers having larger effects than others (Park and Casella 2008); the prior distribution of *α* is not considered normally distributed in BL; *α* is the corresponding vector of marker effects assigned double-exponential (Pérez and de los Campos 2014). As for BRR, the prior assumption is Gaussian. Bayesian methods were conducted by the “BGLR” statistical package in R (Pérez and de los Campos 2014). The default parameters for prior specification were used, and models were trained for 12,000 iterations for the MCMC algorithm with a burn-in of 2000.

In the rrBLUP model, assuming that all marker effects have equal variance with zero covariance, markers were considered random effects. In theory, rrBLUP and GBLUP are equivalent in estimating the variance components. rrBLUP is an efficient solution for the multicollinearity problem, which is a penalized regression-based approach. Models for rrBLUP were implemented using “rrBLUP” packages in the R program (Endelman 2011).

RKHS is a kernel approach that uses a kernel function to convert the marker dataset into a set of distances between pairs of observations, which results in a square matrix and enables regression in a higher-dimensional feature space (Gianola et al. 2006). Here, we implemented the method in the R package BGLR. The model of RKHS is represented as

$$y = 1\mu +{\sum }_{l=1}^{L}{\mathit{u}}_{l}+\varepsilon ,$$

with $p(\mu ,{\mathit{u}}_{1 },...,{\mathit{u}}_{L},\upvarepsilon )\propto {\prod }_{l=1}^{L}N({\mathit{u}}|0,{K}_{l}{\sigma }_{\mathit{u}l}^{2})N(\upvarepsilon$|0,I${\sigma }_{\varepsilon }^{2}$), multi-kernel RKHS defines the sequence of kernels based on a set of reasonable values of *h*; a grid of values of *h* was required by the CV approach to fitting models. *K*_*l*_ is the reproducing kernel evaluated at the *l*th value of the bandwidth parameter in the sequence {*h*_*1*_, …, *h*_*L*_}. The reproducing kernel is one of the central elements of model specification, providing the regression function.

### Genome-wide association study

Data analyzed by GWAS was implemented by GAPIT (version 3) (Wang and Zhang 2021). Manhattan and quantile–quantile plots were implemented with the “qqman” packages in the R program (Turner 2018). For each trait, six independent association-mapping analyses were run using the models of BLINK, Compressed Mixed Linear Model (CMLM), FarmCPU, General Linear Model (GLM), MLM, and SUPER. All GWAS experiments were implemented with accessions that were not included in the corresponding model training process for the genomic prediction experiments. The objective was to avoid the overfitting of the training model.

### Weight model

The generation of the weight model consists of three phases: GWAS results obtained, weight matrix generated, and final modeling based on rrBLUP. The weighting strategy is based on the principle of giving an individual a large weight to loci with large effects and relatively smaller and uniform weights to others. We implemented the approach by the following steps: (1) run GWAS using the method mentioned above, and the GWAS results file consists of a list of marker names with *P*-values; (2) according to the GWAS results under optimal combination, a weight coefficient matrix ω was generated by extracting *P*-values, G corresponding to represent the SNP genotypes coding matrix. The calculation of weighted-matrix W was obtained by as follows: *W* = (1 − ω)*G*; (3) proceed with model solving and validation phenotype prediction using W-matrix obtained in (2).

### Cross-validation

K-fold CV was employed using *k* = 5 to evaluate the predictabilities of the population. The training set was randomly separated into five subsets of the same size and repeated nine times. For each CV experiment, four of the five subsets were used as the training set; the correlation between the predicted and the observed phenotype was calculated. Goodness of fit (GOF) and predictability were adopted to evaluate the effects of traits. GOF is Pearson’s correlation coefficient between the true phenotypes and fitted values of individuals in the training set, and the predictability is the squared correlation between the true phenotypes and estimated phenotypes of individuals in the testing set.

### Simulation design

Different numbers of marker subsets across the genome that consisted of 3955, 3000, 2000, and 1000 markers were randomly generated for simulations, and prediction accuracies across 72 iterations of a fivefold CV scheme for each marker set were averaged. As for the GWAS-GS method, the selection criterion was the association degree of SNP loci with the target phenotypic trait, as measured by GWAS. The top 50, 100, and 200 markers sorted by *P*-values (low to high) were chosen; these associated markers from six GWAS model results were used to build predicted models. Based on marker number 3955, CV folds of 5, 10, and 20 were performed to evaluate their effects on the prediction accuracy. The effect of the environment was separated into three components: the impact of the HZ and LS environment and the multi-environment model combining the phenotypic values of these two locations by BLUE. In addition, an evaluation of GWAS accuracy on the six models of phenotype prediction accuracy was carried out.

## Results

### Prediction performance of traits using genomic selection models

The phenotype of 459 diverse rice varieties in two environments was collected, including HZ environment and LS environment. Nine agronomic traits prediction was performed by the genotypic dataset containing 3955 markers. A total of eight statistical GS models were used for the simulation, and whole-genome prediction models were built by fitting effects for all SNPs. Results in terms of fitting were reported in Table 1, suggesting that the GOF ranged from 0.7702 to 0.9757, which were all relatively high. RKHS provided the highest average GOF (0.9304), whereas BL performed worst (0.8819).

**Table 1:** Goodness of fit and the standard error (SE) using eight genomic selection (GS) models for the nine traits

Trait	GBLUP		rrBLUP		RKHS		BayesA		BayesB		BayesC		BL		BRR	
	Mean	SE	Mean	SE	Mean	SE	Mean	SE	Mean	SE	Mean	SE	Mean	SE	Mean	SE
TN	0.8658	0.0031	0.8637	0.0036	0.9238	0.0036	0.8629	0.0040	0.8635	0.0025	0.8588	0.0030	0.8519	0.0045	0.8670	0.0037
TA	0.7856	0.0061	0.7949	0.0024	0.8812	0.0071	0.7940	0.0032	0.8077	0.0069	0.7856	0.0063	0.7702	0.0076	0.7920	0.0030
PH	0.9718	0.0009	0.9757	0.0008	0.9729	0.0015	0.9644	0.0012	0.9652	0.0007	0.9642	0.0011	0.9668	0.0009	0.9655	0.0016
FLL	0.8869	0.0026	0.8871	0.0026	0.9309	0.0042	0.8812	0.0034	0.8902	0.0022	0.8900	0.0023	0.8840	0.0043	0.8947	0.0030
FLW	0.9263	0.0021	0.9337	0.0032	0.9427	0.0037	0.9320	0.0015	0.9241	0.0031	0.9258	0.0020	0.9293	0.0021	0.9249	0.0021
FLLW	0.9168	0.0023	0.9187	0.0023	0.9423	0.0022	0.9104	0.0028	0.9128	0.0019	0.9112	0.0018	0.9097	0.0027	0.9191	0.0021
FLA	0.8876	0.0026	0.8916	0.0032	0.9373	0.0029	0.8658	0.0248	0.8856	0.0028	0.8878	0.0025	0.8768	0.0054	0.8879	0.0039
PN	0.8913	0.0052	0.8840	0.0075	0.9196	0.0085	0.8887	0.0037	0.8957	0.0033	0.8821	0.0077	0.8799	0.0055	0.8781	0.0079
PL	0.8779	0.0037	0.8740	0.0043	0.9225	0.0024	0.8717	0.0032	0.8734	0.0028	0.8749	0.0029	0.8684	0.0046	0.8720	0.0043
Mean	0.8900	0.0032	0.8915	0.0033	0.9304	0.0040	0.8857	0.0053	0.8909	0.0029	0.8867	0.0033	0.8819	0.0042	0.8890	0.0035

The nine traits were labeled as TN, tiller number; TA, tiller angle; PH, plant height; FLL, flag leaf length; FLW, flag leaf width; FLLW, the ratio of flag leaf length and flag leaf width; FLA, flag leaf angle; PN, panicle number; and PL, panicle length. The eight statistical models were GBLUP, genomic best linear unbiased prediction; rrBLUP, ridge regression best linear unbiased prediction; RKHS, reproducing kernel Hilbert space; BayesA; BayesB; BayesC; BL, Bayesian LASSO; and BRR, Bayesian ridge regression

**Fig. 1:**
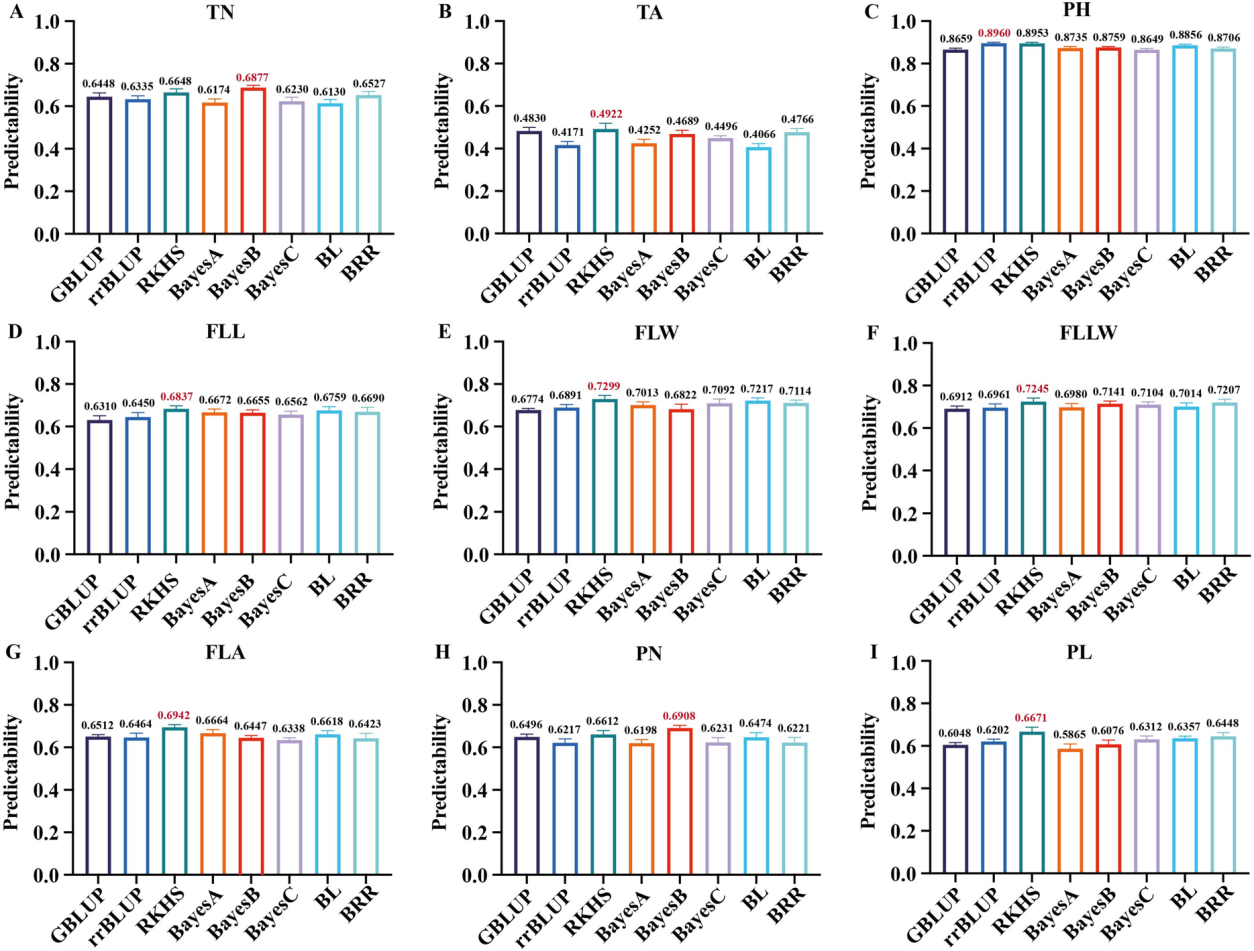
Predictability of the nine traits across eight GS models. Predictability was measured as the Pearson correlation coefficient between predicted and phenotypic values. (**A**–**I**) Predictability of TN, TA, PH, FLL, FLW, FLLW, FLA, PN, and PL, respectively. The *y*-axis indicated the predictability, above and below, and the *x*-axis represented the traits and selection models, respectively. The highest correlation rates for each phenotype were shown in red

GOF measures the fitting ability of the established model to the data. A fitted model represents the reliability of the prediction, while predictability reflects the predictive performance. The predictabilities evaluated by the fivefold CV were presented in Fig. 1. Results showed that the predictabilities of nine traits varied from 0.4066 to 0.8960; the average predictive ability was 0.6629 (across all traits). Additionally, BayesB was the most efficient model for TN (0.6877) and PN (0.6908) (Fig. 1A, H). PH was more accurately predicted with the rrBLUP model (Fig. 1C). Overall, the most successful model was RKHS; it was the top performer in the other six traits. Three traits including FLL, FLW, and FLLW were poorly predicted by GBLUP with accuracies of 0.6310, 0.6774, and 0.6912, respectively.

### Factors affecting genomic prediction

**Fig. 2:**
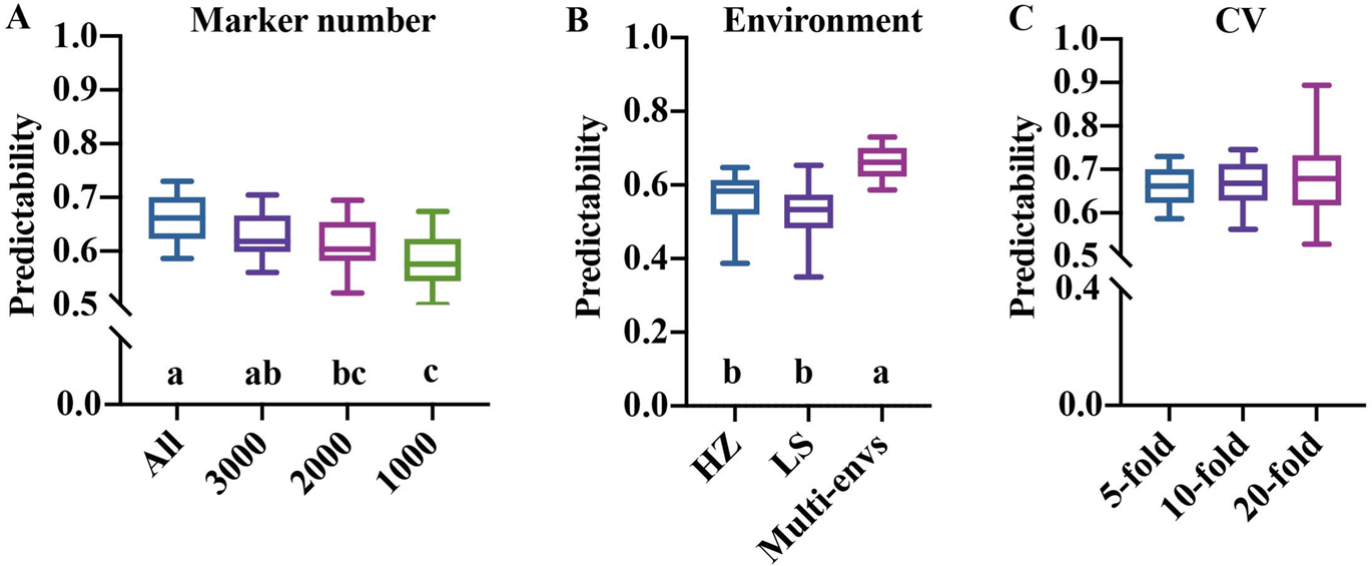
Comparison of the predictive ability with different conditions. Effect of marker number (**A**), environment (**B**), and cross-validation fold (**C**) on the predictive ability of genomic prediction. For each box, the median (horizontal bar) values were represented. Multiple comparisons were conducted using least significant difference (LSD) test (*P* < 0.05). Significant differences between groups were determined by different letters. ns, not significant

The relevant attributes (markers, environment, etc.) have important impacts on the ability of phenotype prediction. To determine the capacity of marker density in prediction accuracy by applying GS in this rice population, a series of different numbers of SNP subsets from 3955 to 1000 across the whole genome were selected. As the number of SNP decreased, the prediction ability decreased significantly from 0.6629 to 0.5813 on average (Fig. 2A). Moreover, the environment was an important factor affecting the prediction accuracy. It was found that the multi-environment model significantly outperformed the single-environment approach in terms of predictability. The average prediction abilities of HZ-, LS-, and multi-environment were 0.5684, 0.5391, and 0.6629, respectively (Fig. 2B). In addition, the analysis of different CV folds on the predictive ability was studied (Fig. 2C). It was shown that the 20-fold CV method (0.6745) barely outperformed tenfold CV (0.6664) and the fivefold CV method (0.6629). In general, the predictability of the three kinds of CV folds varied greatly, but there is not much difference on average. All the comparisons were based on the predicted results of all the models and traits.

### Evaluation of predictabilities using genome-wide association study for implementing genomic selection

We investigated the possibility of improving the prediction performance by incorporating GWAS into GS for different traits applied to the rice dataset. Manhattan plots of all agronomic traits using the six GWAS models were shown in Supplementary Fig. S1.

We first calculated GOF for each trait by applying these GWAS models to the population (Table 2). GOF is an important statistical indicator to test the fitting status of the established model and observation, verifying the expression ability of the model. Among these models, the lowest value of GOF was obtained under GLM with the value of 0.7912 (on average across variables), while the highest value of GOF was 0.9130 by using MLM. As for different traits, PH has the highest GOF (0.9399), followed by FLW (0.8829), FLLW (0.8803), and FLA (0.8757).

**Table 2:** Goodness of fit and SE of the nine traits using genome-wide association analysis (GWAS)-based GS

Trait	BLINK		CMLM		FarmCPU		GLM		MLM		SUPER	
	Mean	SE	Mean	SE	Mean	SE	Mean	SE	Mean	SE	Mean	SE
TN	0.8902	0.0071	0.9112	0.0054	0.8945	0.0069	0.7730	0.0186	0.9107	0.0053	0.8255	0.0130
TA	0.8343	0.0099	0.8345	0.0108	0.8501	0.0089	0.7113	0.0213	0.8711	0.0074	0.7622	0.0163
PH	0.9515	0.0035	0.9603	0.0031	0.9546	0.0034	0.9053	0.0085	0.9602	0.0029	0.9074	0.0080
FLL	0.8715	0.0096	0.9058	0.0067	0.8784	0.0093	0.8060	0.0169	0.9066	0.0066	0.8042	0.0174
FLW	0.9261	0.0049	0.9056	0.0077	0.9062	0.0069	0.7982	0.0201	0.9228	0.0061	0.8383	0.0145
FLLW	0.8916	0.0080	0.9307	0.0050	0.8980	0.0078	0.8184	0.0169	0.9313	0.0048	0.8117	0.0176
FLA	0.8734	0.0101	0.9072	0.0069	0.9054	0.0071	0.8230	0.0160	0.9151	0.0066	0.8301	0.0147
PN	0.8671	0.0096	0.9063	0.0063	0.8903	0.0078	0.7378	0.0222	0.9033	0.0070	0.8025	0.0152
PL	0.8742	0.0081	0.8861	0.0076	0.8750	0.0084	0.7479	0.0211	0.8958	0.0067	0.7590	0.0204
Mean	0.8867	0.0079	0.9053	0.0066	0.8947	0.0074	0.7912	0.0180	0.9130	0.0059	0.8157	0.0152

The six GWAS models were BLINK, Bayesian information and Linkage-disequilibrium Iteratively Nested Keyway; CMLM, Compressed Mixed Linear Model; FarmCPU, Fixed and random model Circulating Probability Unification; GLM, General Linear Model; MLM, Mixed Linear Model; SUPER, Settlement of MLM Under Progressively Exclusive Relationship

**Fig. 3:**
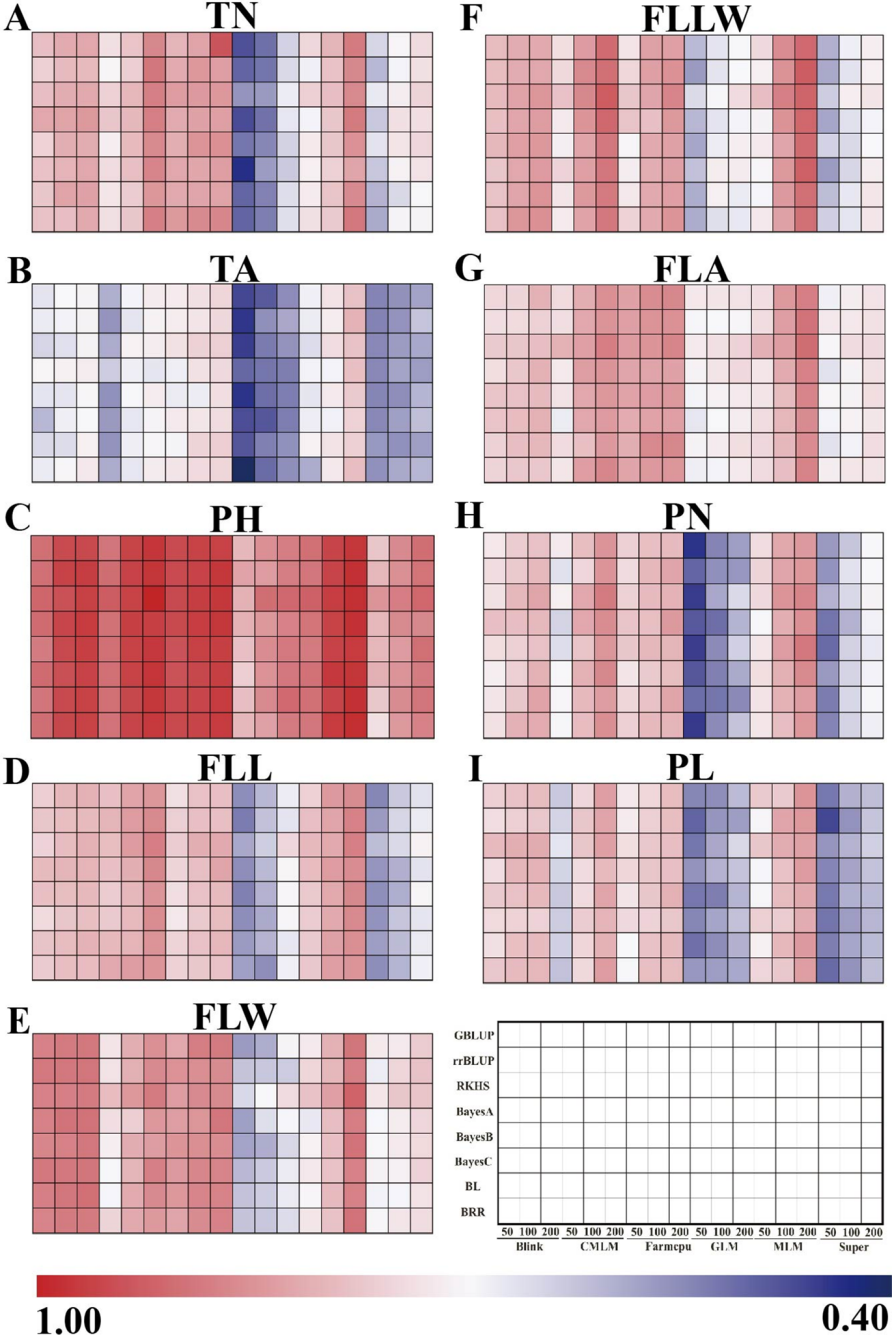
Heat maps of predictive abilities of the nine traits from GWAS-GS methods. Predictions varied in the number of SNPs, GWAS and GS models, and traits. The rankings were shown as different shades from blue to red

The predictability values of the eight GS models for the nine traits under six GWAS models were transformed to generate a heat map (Fig. 3); the strength of the prediction precision is given by that. Detailed data were supplied in the Supplementary Table. S1. The models for prediction were conducted on all three marker sets (top 50, 100, and 200 of GWAS-SNPs) selected by the six GWAS models’ the results showed a moderate-to-high prediction ability, ranging from 0.4711 to 0.9358. The predictive ability of TA, FLW, FLA, PN, and PL were the highest in the MLM-rrBLUP, MLM-RKHS, MLM-RKHS, MLM-BayesB, and MLM-GBLUP combinations by using GWAS top-200 SNP subsets, and the values were 0.7822, 0.8720, 0.8607, 0.8493, and 0.8165, respectively. Moreover, PH, FLL, and FLLW gained the best predictive performance by the CMLM-RKHS model (0.9358, 0.8409, and 0.8806, respectively). TN had the largest span of predictive accuracy, varying from a minimum of 0.4976 (GLM-BayesC) to a maximum of 0.8868 (FarmCPU-GBLUP). However, models that used the top-50 SNP subsets from GLM got the worst predictabilities in TN, TA, FLW, PN, FLL, and FLLW (0.4976, 0.4711, 0.5998, 0.5107, 0.5686, and 0.5996, respectively); SUPER underperformed with other three traits.

### Multiple comparisons of predictabilities based on GWAS-GS

**Fig. 4:**
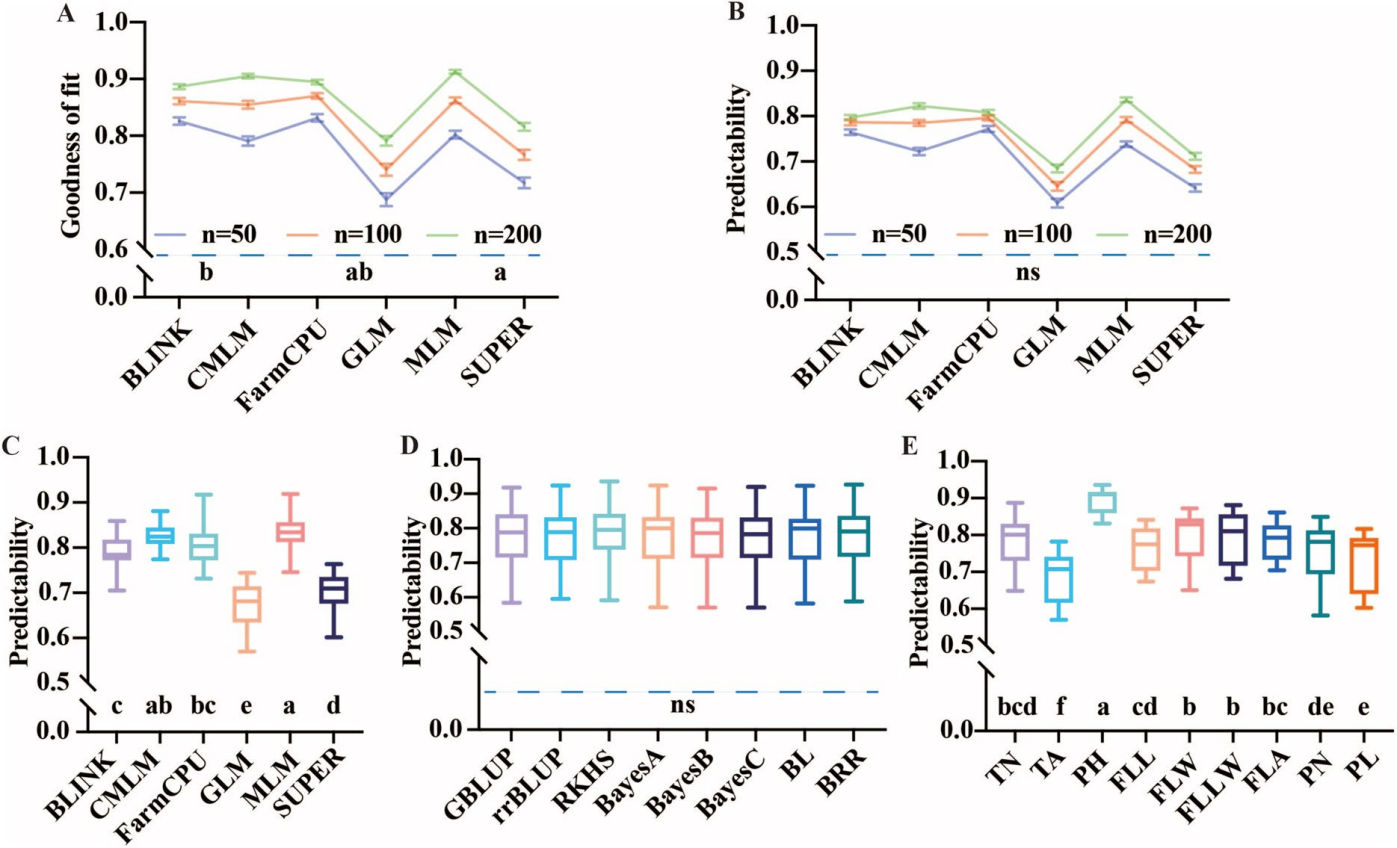
Multiple comparisons of predictabilities illustrated by boxplots. Comparison of goodness of fit (**A**) and predictive ability (**B**) with GWAS top 50, 100, and 200 maker sets. (**C**) Comparison of predictabilities for the six GWAS models over eight GS models and the nine traits. (**D**) Comparison of predictabilities of the eight GS models across six GWAS models and the nine traits. (**E**) Comparison of predictabilities of the nine traits over six GWAS models and eight GS models. Multiple comparisons were conducted using least significant difference (LSD) test (*P* < 0.05). In each panel, different lowercase letters below the group labels representing significant differences between groups. ns, not significant

We made an analysis of variance for the predictabilities of 9 × 6 × 8 = 432 traits-GWAS_model-GS_model combinations. All main effects and interaction effects were significant (Supplementary Table S2). Multiple comparisons were conducted to reveal the factors that affected the prediction accuracy, such as the marker density, the traits, GWAS models, and the statistical methods (Fig. 4).

The mean GOF was 0.7758, 0.8258, and 0.8678 for top 50, 100, and 200 associated markers, respectively (Fig. 4A). Although there was no significant difference among the predictive accuracy of GWAS top marker sets, under MLM, the top-200 marker set was more feasible for data analysis (Fig. 4B). The predictive accuracy was 0.7079, 0.7480, and 0.7767 for top 50, 100, and 200 associated markers, respectively. By comparing various GWAS models, it displayed that these models were classified into five significance levels of predictability. The average prediction accuracies are 0.7968, 0.8226, 0.8083, 0.6849, 0.8361, and 0.7115 for BLINK, CMLM, FarmCPU, GLM, MLM, and SUPER, respectively. It demonstrated that the average accuracies of MLM, CMLM, and FarmCPU were better than other GWAS models (Fig. 4C). What is more, it was found that all the eight GS models achieved comparable prediction abilities (Fig. 4D). However, the accuracy of RKHS consistently outperformed others relatively. The average prediction accuracies are 0.7892, 0.7771, 0.7765, 0.7754, 0.7752, 0.7745, 0.7735, and 0.7722 for RKHS, GBLUP, BayesA, BRR, rrBLUP, BayesB, BL, and BayesC, respectively. Not much variation among different GS models was observed, and the differences exceeded no more than 2%. In addition, multiple comparisons between GWAS-GS combinations were provided in Supplementary Table S3. Furthermore, the prediction accuracy of nine traits was compared and classified into six significance levels (Fig. 4E). The most predictable traits in rice were PH (0.8935), FLW (0.7985), and FLLW (0.7935). TA exhibited a relatively low Pearson’s correlation coefficient value of 0.6871.

### Genomic prediction from GWAS-based weighted-matrix in rice

**Fig. 5:**
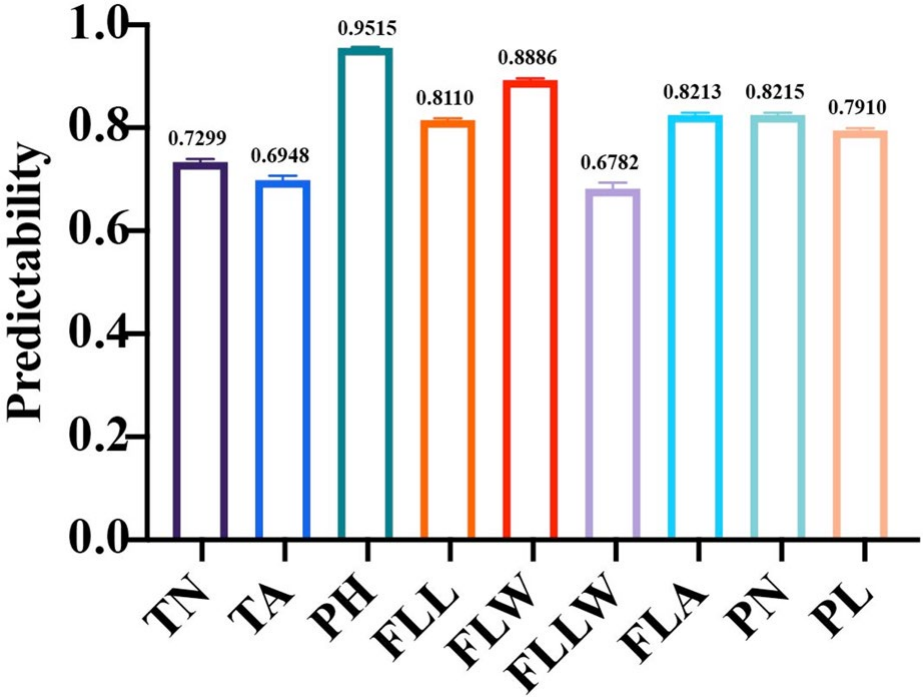
Predictabilities from the weighted matrix using the rrBLUP model

According to the results described above, we found that integrating GWAS and GS might be a feasible approach. Thus, a further exploration of evaluating the potential predictive ability for using GWAS was taken. We proposed a method of utilizing the most accurately predicted GWAS model and then constructed a weight matrix using the *P*-values of every marker in GWAS results to optimize rrBLUP. The GWAS results used for building models were derived from the performance in Fig. 3 in each trait (MLM for TA, FLW, FLA, PN, and PL; CMLM for PH, FLL, and FLLW; FarmCPU for TN). The increase in predictability was larger for low predictability traits (Fig. 5). Comparison was made based on the average predictive of nine traits from Fig. 1 (0.6421, 0.4524, 0.8785, 0.6617, 0.7028, 0.7071, 0.6551, 0.6420, and 0.6247 for TN, TA, PH, FLL, FLW, FLLW, FLA, PN, and PL, respectively). Based on the weighted-matrix rrBLUP model, the predictive ability of TA increased by 24.244%. While the average performance for FLW, PN, PL, FLA, and FLL performed similarly by a raise of 16.942%. However, FLLW showed a slight trend of downward, with a decrease of no more than 2.9%. Overall, applying this approach can be an alternative way to predict low predictability traits in rice.

## Discussion

### Comparison of genomic selection models

GS has shown its potential in plant breeding during the last two decades. It has been widely used in the phenotypic prediction of crops (de Oliveira et al. 2012; Rutkoski et al. 2015; Xavier et al. 2016). GS algorithm plays a vital role in prediction; its predictability varies with assumptions and treatment of marker effect. Therefore, significant differences may occur among different GS models, even under the same trait (Onogi et al. 2015). In our study, out of the eight GS models we studied, RKHS achieved the best performance across six of the total nine traits, whereas GBLUP and BL were found to be the least predictive (Fig. 1). Similarly, compared with other GS models for genomic prediction, RKHS was proven to be the preferable model across different species (Heslot et al. 2012; Hong et al. 2020). As for TN and PN, BayesB was the most efficient model in our dataset; however, from the comparison of different prediction models, Xu et al. (2017) found that BayesB was the overall worst performer. Hence, one lesson that can be learned from applying a variety of models to different datasets is that no single model can universally well predict all the data.

### Effect of marker density, environment, and training set size on predictability

Typically, high-density marker sets were ideal for enhancing the prediction accuracy. This situation is consistent with our study; the average predictive ability of all 3000, 2000, and 1000 marker models were 0.6629, 0.6286, 0.6108, and 0.5813, respectively, which showed three levels of significance (Fig. 2A). The all-marker model gave the highest predictive accuracy, which was significantly higher than others. However, studies have found that an appropriate number of markers can reduce the dimensionality of genomic data, subsequently reducing the size of the parameters pool and allowing a better generalization of the predictive model. This has been proven in the study by Xu et al. (2018); the prediction accuracy plateaued when 5000 markers were applied in the model. Given these points, it is always difficult to give a benchmark for the number of markers to be used in GS.

Phenotype variation is derived from genotype-by-environment interaction. We performed CV using phenotype data collected at two locations (HZ and LS). The accuracy for multi-environment prediction (0.6629) was higher than those for single-environment at 0.5684 and 0.5391 on average across all traits. This is consistent with what would be expected by theory. In the same way, gains from multi-environment models have been found for a variety of crops, including rice and wheat (Cuevas et al. 2016; Sukumaran et al. 2018; Bhandari et al. 2019).

In addition to the factors above, predictability can also be affected by variables such as heritability and the size of the training set (Spindel et al. 2015; Ben Hassen et al. 2018). In this study, different numbers of samples were classified by different CV folds. According to our dataset, stochastic simulations were conducted in which the CV folds varied among 5, 10, and 20, respectively. The increase in CV folds was accompanied by the raised number of groups divided into the training set, causing a range of predictive accuracy from 0.6629 to 0.6745 (Fig. 2C). This is compatible with Wang et al. (2015), who found that the prediction ability for a two-fold CV was much lower than that for a ten-fold CV. The accuracy was decreased from 0.259 to 0.206, with the training population size varied from 539 to 300.

### The feasibility of using GWAS-GS models to predict traits in plants

Further investigation of prediction methods is crucial for the accurate prediction of plant phenotypes; researchers are committed to optimizing the algorithms and exploring the conditions (Zhao et al. 2013). New strategies for efficient selection, for instance, incorporating parental phenome or using omics data for model building, brought a distinct benefit for prediction in crop breeding (Xu et al. 2017; Xu et al. 2021). In addition to these strategies, it has been investigated whether approaches to integrating GWAS with GS are feasible.

GWAS have enabled the dissection of the genetic architecture of complex traits in more than a dozen plants (Zhu et al. 2008). In 2010, the first rice GWAS study was performed (Huang et al. 2010); since then, GWAS has been successfully used in rice for more than 10 years, revealing a great number of loci that are associated with crucial agronomic traits (Wang et al. 2020). If one model corresponds to the genetic architecture of a trait, this model will get high prediction accuracy. Therefore, using GWAS to estimate marker importance value or screen the SNPs that are significantly related to traits could increase the trait predictability in GS (Zhang et al. 2023; Singer et al. 2022). In addition, GWAS approaches reduce the dimensionality of genomic data for prediction and decrease the computational burden. Also, avoiding capturing numerous false genotype–phenotype relationships or building less discriminant genomic distances enabled us to improve accuracy with non-redundant SNP matrices. Lastly, it provides a small number of model parameters for better generalization in prediction modeling (Bermingham et al. 2015). Studies have manifested the utility of the application of GWAS on prediction problems (He et al. 2019; Singer et al. 2022). Using the selected markers obtained via GWAS at different levels of *P*-values, six traits were successfully predicted using BLUP in maize (Xu et al. 2017).

### Applications of GWAS-associated markers to GS

**Fig. 6:**
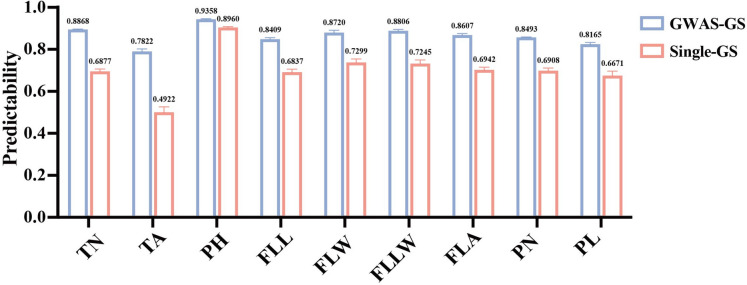
Comparison of predictabilities between single-GS and GWAS-GS model

The joint analysis between GS and multi-GWAS models was investigated in our work. The optimal model obtained may change as the predictor variable (Rakotoson et al. 2021). Therefore, to make the results representative and practical, we utilized a total of 432 traits-GWAS_model-GS_model combinations in the same genetic background for prediction (Fig. 3). The accuracy for the GWAS joint model, on average, performed the best (MLM) of 0.8361 across all traits (Fig. 4). While the model built on GLM was the worst, the accuracy of CMLM, FarmCPU, and BLINK was in the middle of MLM and SUPER, indicating MLM may have a better ability to select SNPs that has a strong benefit with traits in this population. GWAS-GS approaches were shown to improve the accuracy of predictions over the classical GS, suggesting that it is not the marker number but the number of useful markers that determines accuracy for predicted traits. The improvement over the model ranged from 3.98 to 29%. The prediction of all traits was leveled up by the GWAS-GS method, and the improvements of TN, TA, PH, FLL, FLW, FLLW, FLA, PN, and PL were 19.91%, 29%, 3.98%, 15.72%, 14.21%, 15.61%, 16.65%, 15.85%, and 14.94%, respectively (Fig. 6). The GWAS-GS results used for comparison were derived from the best predictive performance of each trait in Fig. 3, and the single-GS results used for comparison were the best prediction results of each trait in Fig. 1. Altogether, the interaction between GWAS and GS indeed has the ability to achieve better predictions than the single-GS (using total SNPs without processing). And we recommended the combination of RKHS-MLM for prediction in rice.

### The possibility of improving accuracy in weighted ways

How to effectively incorporate GWAS results into the prediction method is obviously a momentous issue. The study has found that it may produce a genetic gain by a weighted approach. Zhang et al. (2014) chose the corresponding marker weights by *T* = ω*S* + (1 − ω)*G*. Here, $S=\frac{{M}_{1}\mathrm{diag}({h}_{1},...{h}_{m1}){\mathrm{M}}_{1}^{\mathrm{T}}}{\mathrm{c}1}$, $\omega$ represented weight, and *h*_1_, *h*_2_, …, *h*_*m*1_ are certain marker weights which have to be obtained beforehand. *G* corresponds to the standard genomic relationship matrix, and the classification of the markers to *M*_1_ was based on GWAS results; this helped to account for relatively more crucial regions across the whole genome and improved the accuracies. In the last section of our study, we aimed to develop a useful approach by taking advantage of the weight matrix generated from the GWAS result. When the weight matrix performed on the training set was implemented in the rrBLUP model, the prediction ability was improved for eight out of the nine agronomic traits (Fig. 5), suggesting that extreme care would need to be taken if perusing this strategy in rice populations. Indeed, this remains a relatively unexplored area of research, leaving for further studies.

## Conclusion

In summary, we used a rice population consisting of 459 varieties to evaluate the factors affecting prediction. The results demonstrated that predictabilities vary in methods, with the RKHS and BayesB models being more recommended than the others. The number of markers and environment had a great influence on prediction. Moreover, a combination of GWAS and GS was also evaluated as a potential method for leading to a substantial accuracy increase in traits; combining MLM and RKHS seems to be a highly effective choice for prediction. We hope that these results obtained in the present study can benefit breeders in selecting ideal varieties, guiding the development of a new transformative strategy for rice improvement.

### Supplementary Information

Below is the link to the electronic supplementary material.

Supplementary file1 (XLSX 30 KB)

Supplementary file2 (DOCX 12 KB)

Supplementary file3 (XLSX 10.7 KB)

Supplementary file4 (JPG 17 MB)

## Data Availability

The datasets analyzed during the current study can be freely and openly accessed at https://datadryad.org/stash/dataset/doi:10.5061/dryad.cp25h. Moreover, the relevant raw data supporting the conclusions of this study are included within the article and within its supplementary materials.

## Abbreviations

*GS*: Genomic selection
*GEBV*: Genomic-estimated breeding values
*RKHS*: Reproducing kernel Hilbert space
*GWAS*: Genome-wide association analysis
*MLM*: Mixed Linear Model
*FarmCPU*: Fixed and random model Circulating Probability Unification
*CMLM*: Compressed Mixed Linear Model
*rrBLUP*: Ridge regression best linear unbiased prediction
*MAS*: Marker-assisted selection
*QTL*: Quantitative trait locus
*BLUP*: Best linear unbiased prediction
*LASSO*: Least absolute shrinkage selection operator
*RF*: Random forest
*SVM*: Support vector machine
*GBLUP*: Genomic best linear unbiased prediction
*ssGBLUP*: Single-step genomic best linear unbiased prediction
*G matrix*: Genomic relationship matrix
*BL*: Bayesian LASSO
*BRR*: Bayesian ridge regression
*CV*: Cross-validation
*SNP*: Single nucleotide polymorphism
*SUPER*: Settlement of MLM Under Progressively Exclusive Relationship
*BLINK*: Bayesian information and Linkage-disequilibrium Iteratively Nested Keyway
*HZ*: Hangzhou
*LS*: LingShui
*TN*: Tiller number
*TA*: Tiller angle
*PH*: Plant height
*FLL*: Flag leaf length
*FLW*: Flag leaf width
*FLLW*: The ratio of flag leaf length and flag leaf width
*FLA*: Flag leaf angle
*PN*: Panicle number
*PL*: Panicle length
*BLUE*: Best linear unbiased estimate
*MCMC*: Markov chain Monte Carlo method
*GLM*: General Linear Model
*GOF*: Goodness of fit
