# Supplementary material for: Integrating genome-wide association study into genomic selection for the prediction of agronomic traits in rice (Oryza sativa L.)
Source: Mol Breed. 2023 Nov 13;43(11):81. doi: 10.1007/s11032-023-01423-y (PMC10641074; doi:10.1007/s11032-023-01423-y)
Supplement: Supplementary file 2 — Supplementary file2 (DOCX 12 KB) [file 11032_2023_1423_MOESM2_ESM.docx]

**Table S2 Analysis of variance of predictabilities from a 9×6×8 factorial design with nine traits, six GWAS models, and eight GS models.**

| **source** | **Df** | **Sum of square** | **Mean square** | ***F*-value** | ***P*-value** |
| --- | --- | --- | --- | --- | --- |
| **Traits** | **8** | **1.1926** | **0.14907** | **1201.454** | **< 2e-16** |
| **GS_model** | **7** | **0.0106** | **0.00151** | **12.166** | **1.43e-13** |
| **GWAS_model** | **5** | **1.4205** | **0.28411** | **2289.727** | **< 2e-16** |
| **GWAS_model:GS_model** | **35** | **0.0109** | **0.00031** | **2.508** | **1.81e-05** |
| **Traits:GS_model** | **56** | **0.0097** | **0.00017** | **1.393** | **0.0444** |
| **Traits:GWAS_model** | **40** | **0.1411** | **0.00353** | **28.439** | **< 2e-16** |
| **Residuals** | **280** | **0.0347** | **0.00012** |  |  |
