# Supplementary figures and images for: Integrating genome-wide association study into genomic selection for the prediction of agronomic traits in rice (Oryza sativa L.)
Source: Mol Breed. 2023 Nov 13;43(11):81. doi: 10.1007/s11032-023-01423-y (PMC10641074; doi:10.1007/s11032-023-01423-y)

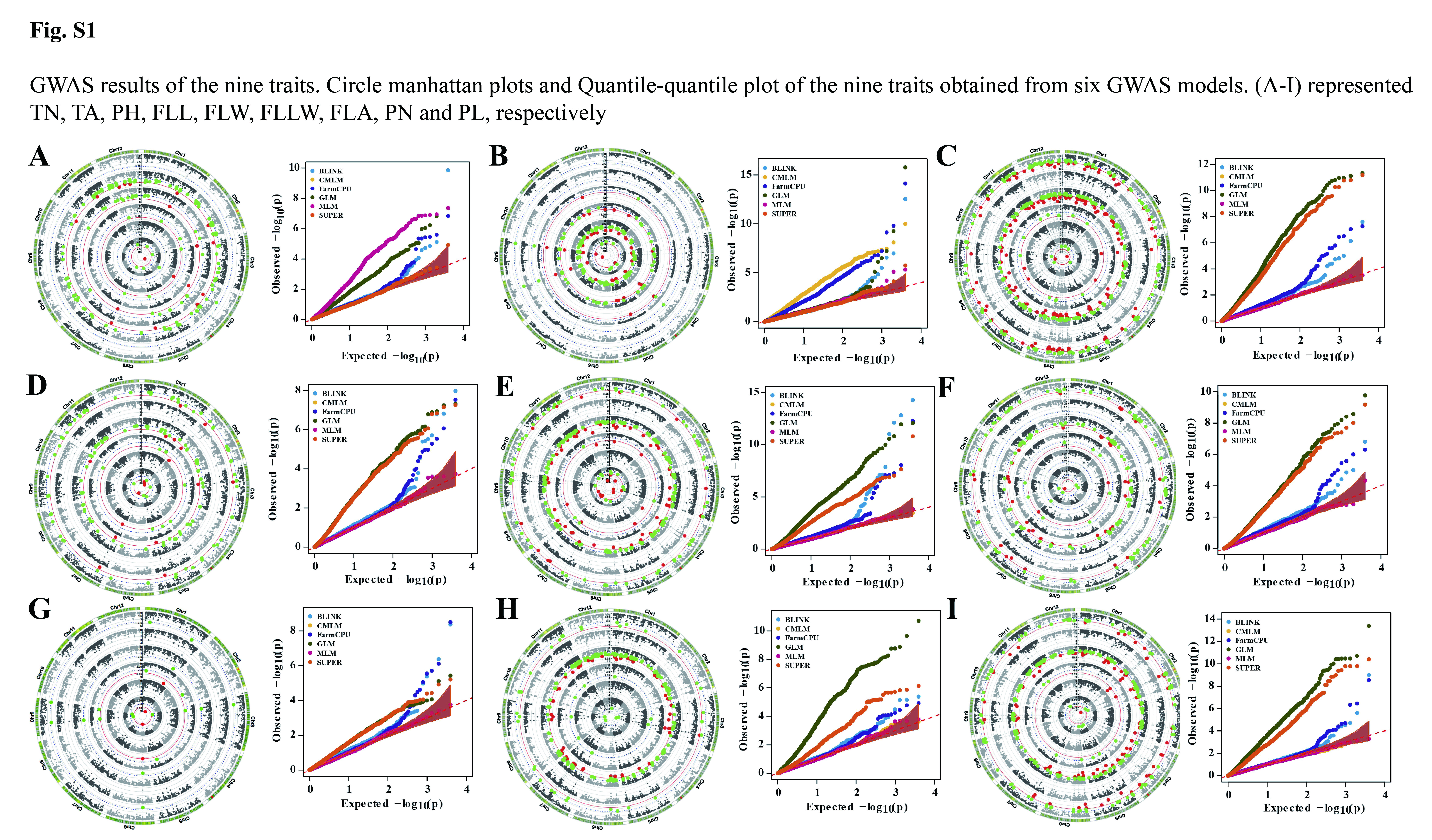

Supplement: Supplementary file 4 — Supplementary file4 (JPG 17 MB) [file 11032_2023_1423_MOESM4_ESM.jpg]
